# N-Oxalylglycine-Conjugated Hyaluronic Acid as a Macromolecular Prodrug for Therapeutic Angiogenesis

**DOI:** 10.3390/gels11010027

**Published:** 2025-01-02

**Authors:** Andrew H. DeMaria, Jeoung Soo Lee, Ken Webb

**Affiliations:** 1 Microenvironmental Engineering Laboratory, Department of Bioengineering, Clemson University, Clemson, SC 29634, USA; ahdemaria1@gmail.com; 2 Drug Design, Development, and Delivery (4D) Laboratory, Department of Bioengineering, Clemson University, Clemson, SC 29634, USA; ljspia@clemson.edu

**Keywords:** hyaluronic acid, macromolecular prodrug, drug delivery, angiogenesis, hypoxia-inducible factor (HIF)-1

## Abstract

Hypoxia-inducible factor-1α (HIF-1α) initiates the cellular response to low oxygen levels, making it an attractive target for stimulating therapeutic angiogenesis. Several small molecules have been identified that stabilize HIF-1α and activate the angiogenic signaling pathway. However, achieving therapeutic doses of bioactive small molecules in target tissues remains challenging. In this paper, we report the synthesis and characterization of a new macromolecular prodrug composed of the pro-angiogenic small molecule N-oxalylglycine conjugated to hyaluronic acid (HA-NOG). NOG was conjugated to HA by esterification, and release was significantly increased in the presence of degradative enzymes, esterase and hyaluronidase, compared to physiological buffer, confirming that the release of NOG is primarily enzymatically driven. Normal human dermal fibroblasts (NHDFs) cultured with HA-NOG exhibited HIF-1α accumulation in the cell nucleus and dose-dependent increases in mRNA expression levels of three direct HIF transcriptional targets. Conditioned medium from these cells stimulated endothelial cell tubulogenesis. As an initial evaluation of safety and possible side effects, HA-NOG was found not to significantly affect NHDF metabolic activity, proliferation, or collagen deposition. These studies demonstrate that HA-NOG releases NOG in response to cellular enzymatic activity, activating the HIF signaling pathway and culminating in the secretion of soluble factors that activate endothelial cells without adversely affecting other cellular metabolic pathways.

## 1. Introduction

Therapeutic angiogenesis seeks to activate and augment the body’s intrinsic mechanisms for creating new microvasculature in order to alleviate tissue ischemia resulting from vascular pathology and traumatic injury [1,2]. Hypoxia-inducible factor-1α (HIF-1α), the heterodimeric master transcription factor of oxygen-sensing pathways, is the key to this process [3,4]. HIF-1α stability and activity are regulated by enzymes that require oxygen (O_2_), iron (Fe^2+^), and 2-oxoglutarate (2-OG). In a normoxic environment, HIF-1α is modified with hydroxyl groups at Pro402/564 by prolyl hydroxylase domain enzymes (PHDs), promoting binding of the von Hippel Lindau (VHL) ubiquitin ligase complex and targeting for proteosomal degradation [5,6,7,8]. In addition, Factor Inhibiting HIF (FIH) can modify HIF-1α with a hydroxyl group at Asn803, blocking its CREB-binding protein (CBP)/p300 nuclear co-activator binding site to prevent transcription [9,10]. In a hypoxic environment, PHDs and FIH are inactivated by lack of O_2_, allowing HIF-1α to translocate to the nucleus, complex with HIF-1β, bind to hypoxia response elements (HRE), and initiate transcription of a wide range of target genes. Among these are numerous pro-angiogenic factors, including vascular endothelial growth factor (VEGF), erythropoietin (EPO), basic fibroblast growth factor (bFGF), and platelet-derived growth factor (PDGF) [11,12]. These growth factors stimulate the formation of new microvessels to alleviate ischemia and support tissue repair.

Delivery of exogenous, pro-angiogenic growth factors is the most widely studied approach to activating therapeutic angiogenesis, particularly VEGF, which is widely recognized for its ability to increase vascular permeability and stimulate endothelial cell proliferation, migration, and tubulogenesis [13]. VEGF injection has been shown to initiate angiogenesis in numerous preclinical models; however, the newly formed microvessels are often leaky, immature, or unstable, and their efficacy in human clinical trials has been limited [14]. One approach to overcome these challenges is the development of improved, biomaterial-based delivery systems that provide spatiotemporal control of VEGF release or sequential release of VEGF and other angiogenic factors such as platelet-derived growth factor (PDGF) that recruits mural cells to stabilize newly formed microvessels (recently reviewed in [15,16]).

Another approach is directly targeting HIF-1α to more broadly activate the angiogenic pathway. Gene therapy using vectors encoding constitutively active forms of HIF-1α has been shown to promote angiogenesis in a variety of tissues [17]. However, clinical translation is hindered by limited control over expression levels and possible detrimental side effects of prolonged, high-level expression of VEGF and other HIF-1 targets [18]. Consequently, small molecule stabilization of HIF-1α is becoming a promising option for targeting the angiogenic pathway. Iron chelators, 2-OG competitive mimics, phenolic compounds, and tricarboxylic acid cycle intermediates have all been shown to inhibit PHD and FIH with varying selectivity, inducing a pseudo-hypoxic response including HIF-1α stabilization and subsequent activation of pro-angiogenic gene expression [19,20,21]. The most widely studied compounds include the iron chelator Desferoxamine (DFO) and the 2-OG mimic N-oxalylglycine (NOG) and its derivative dimethyloxalylglycine (DMOG) that have been shown to increase vascularization and improve healing in diabetic ulcers, bone fractures, and traumatic brain injury [22,23,24,25,26]. Relative to recombinant growth factors, small molecule therapeutics are advantageous due to their stability, cost-effectiveness, and limited immunogenicity. However, delivery still presents some challenges as they are typically distributed systemically and cleared from the body too quickly [27,28] after systemic administration [29,30].

Hydrogels offer many advantages as local drug delivery systems, including their minimally invasive implantation by injection, in situ crosslinking, and ability to support cell adhesion, migration, and cell-mediated remodeling. For small molecule therapeutics, hydrogels must incorporate mechanisms to achieve efficient incorporation of hydrophobic drugs with limited aqueous solubility and overcome burst release of hydrophilic drugs. One approach to improve the solubility and biodistribution of small molecules is their conjugation to polymers to create macromolecular prodrugs. Hyaluronic acid (HA), a naturally occurring glycosaminoglycan (GAG), has been used as a macromolecular backbone for conjugating a wide range of small molecules including taxol, dexamethasone (DX), and curcumin for use in anti-cancer/anti-tumor, anti-inflammatory, and wound healing applications [31,32,33,34]. Recently, we described semi-interpenetrating polymer network hydrogels composed of photo-crosslinked polyethylene glycol diacrylate and DX-conjugated HA as matrices for tissue repair via local drug delivery. These networks have been shown to provide cell-mediated, sustained DX release, support in vitro osteogenesis of encapsulated human mesenchymal stem cells, reduce secondary injury, and improve functional recovery in a rodent traumatic brain injury model [35,36,37,38]. The objective of this study was to develop a macromolecular prodrug for therapeutic angiogenesis. We describe the synthesis and characterization of N-oxalylglycine-conjugated HA (HA-NOG) and show its ability to stabilize HIF-1α, increase transcription of HIF-1α targets, and promote endothelial cell tubulogenesis.

## 2. Results and Discussion

Successful use of bioactive small molecules in regenerative medicine still has several challenges to overcome, most notably limited solubility of hydrophobic drugs and rapid clearance of hydrophilic drugs, making it difficult to achieve a therapeutic dose at the target site while minimizing systemic side effects. The goal of this study was to develop N-oxalylglycine-conjugated hyaluronic acid (HA-NOG) as a macromolecular prodrug that can be incorporated within hydrogels, be released in response to cell-mediated remodeling, and stimulate localized activation of therapeutic angiogenesis.

### 2.1. Synthesis and Characterization of HA-NOG

**Figure 1:**
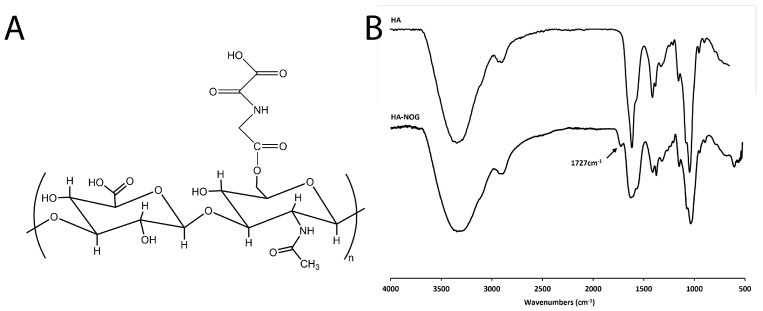
Structure of HA-NOG characterized by ATR-FTIR. (**A**) Predicted structure and (**B**) FTIR spectrum of HA-NOG showing ester carbonyl peak at 1727 cm^−1^ along with peaks originating from HA.

HA-NOG was synthesized by CDI-mediated esterification between the NOG carboxylic acid group and the HA hydroxyl group (Figure 1A).

Using ATR-FTIR, the structure of HA-NOG was verified and compared with native HA (Figure 1B). Both spectra showed the broad O-H stretch for HA from 2800–3600 cm^−1^ and C=O stretch for carboxylic groups at 1609 cm^−1^ consistent with previous FTIR analyses of HA reported in the literature [39,40]. For HA-NOG, the IR spectrum included an ester carbonyl peak at 1727 cm^−1^. We first performed the synthesis at 0.85:1 NOG/HA molar ratio), which resulted in a degree of substitution (DS) of 2.52% (mg drug per 100 mg HA). When the amount of NOG was increased to a 1.7:1 molar ratio, the DS was increased to 3.99%. HA-NOG with 3.99% DS was used for all subsequent studies.

### 2.2. Enzymatic Release of NOG from HA-NOG Conjugate

**Figure 2:**
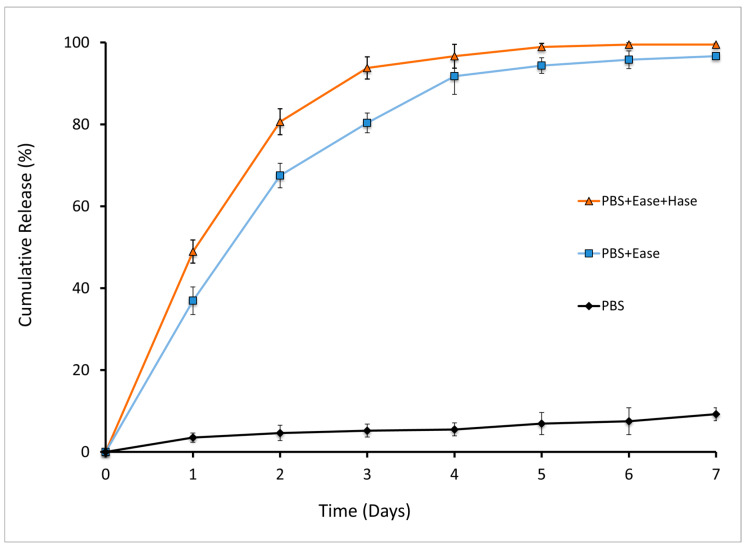
Release of NOG in the absence and presence of Ease and Hase at 37 °C over 7 days.

Release of NOG from HA-NOG was evaluated during chemical and enzymatic hydrolysis. Enzymatic degradation of HA-NOG was expected to be the primary mechanism for NOG release. HA-NOG was incubated in phosphate-buffered saline (PBS), PBS with Ease, and PBS with Ease and Hase for 7 days (Figure 2). Incubation in PBS alone resulted in 7% cumulative NOG release over 7 days, with no significant differences in the amount of NOG released between any days within that span. In the presence of Ease alone and Ease/Hase, cumulative NOG release was significantly increased at all time points relative to PBS alone, with 78% and 90% released by Day 3, respectively, and 93% and 99% released by Day 7. Both conditions resulted in significant increases in released NOG each day from Day 0 to Day 3, while Ease alone also significantly increased release from Day 3 to Day 4. These results demonstrate that despite conjugation through a double ester, HA-NOG is relatively stable in the physiological buffer. HA has commonly been used as a backbone for macromolecular prodrugs in order to increase the aqueous solubility of hydrophobic drugs, target drugs to cancers overexpressing HA receptors, and increase circulation/residence time [41]. The time required for drug release varies from days to weeks, likely depending upon the drug’s physicochemical properties, conjugation chemistry, and adjacent chemical structures, such as spacer groups [32,42,43,44]. Our results demonstrate that HA-NOG is relatively stable in the physiological buffer; this is consistent with previous observations of HA-DXM conjugated in a similar manner [35]. This suggests that when incorporated into hydrogels, HA-NOG will be able to sequester hydrophilic NOG within the network until released in response to cellular enzymatic activity.

### 2.3. Evaluation of Angiogenic Bioactivity

#### 2.3.1. HIF-1α Stabilization

**Figure 3:**
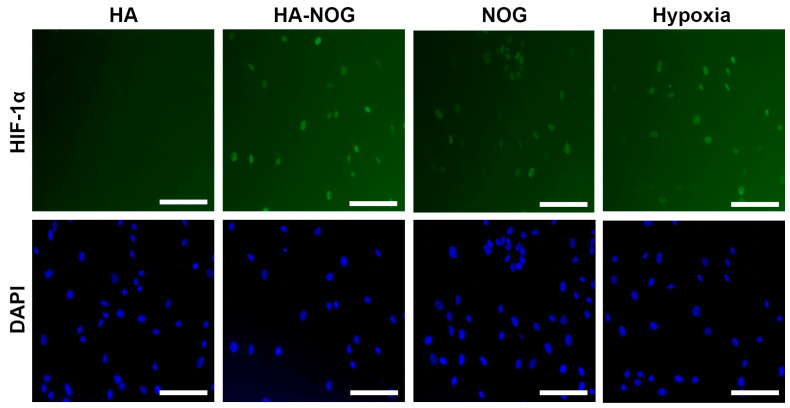
Immunocytochemical analysis of HIF-1α (green) and DAPI nuclear stain (blue) in normal human dermal fibroblasts (NHDFs) cultured with hyaluronic acid (HA, 24 nM), HA with conjugated N-oxalylglycine (HA-NOG, 24 nM), NOG (10 μM), and hypoxia (1% O_2_) at 6 h. Scale bar = 200 μm.

To evaluate the ability of HA-NOG to activate the angiogenic response in target cells, we first investigated its ability to stabilize the HIF-1α subunit that is constitutively degraded under normoxic conditions. HA-NOG was compared to NOG alone (free drug); untreated and native HA as negative controls; and CoCl_2_ and hypoxia as positive controls. An amount of 10 μM NOG was chosen based on previous reports demonstrating that this concentration was effective for inhibition of PHD2 and FIH and stabilization of HIF-1α [21,45]. From the DS of HA-NOG, we calculated that 24 nM HA-NOG would contain 10 μM NOG. HIF-1α protein was undetectable by immuno-staining in normal human dermal fibroblast (NHDF) cultures that were untreated (Figure S1) or exposed to native HA (Figure 3). NHDFs treated with free NOG, HA-NOG, or exposed to hypoxia (Figure 3) or CoCl_2_ (Figure S1) exhibited positive staining for HIF-1α localized to the cell nucleus.

There was no observable difference in HIF-1α expression between HA-NOG and the positive controls, demonstrating the conjugate’s efficacy in stabilizing HIF-1α. Since high molecular weight HA cannot passively cross the cell membrane, there are several possible mechanisms by which NOG may reach the cytoplasm and prevent HIF-1α degradation. One possibility is that esterase enzymes present in the serum-containing culture medium may release NOG, allowing the free drug to diffuse across the cell membrane. Alternatively, HA-NOG may be actively internalized as degradation of high molecular weight HA has been found to involve extracellular cleavage by hyaluronidase 2 followed by CD44-mediated internalization of the resulting ~20 kDa fragments for further lysosomal degradation [46]. The first reports investigating the role of PHDs and FIH in HIF-1α stability demonstrated that NOG inhibited the activity of isolated enzymes but used the more hydrophobic derivative DMOG for cell culture studies [8,9]. This was based upon the notion that DMOG would have superior membrane transport, and although a direct comparison of NOG and DMOG was not reported, DMOG has been used almost exclusively since. Interestingly, in our studies, NOG added to the culture medium as a free drug showed comparable efficacy for HIF-1α stabilization as HA-NOG and the CoCl_2_/hypoxia-positive controls. Therefore, both mechanisms proposed above involving extracellular and intracellular NOG release may contribute to the ability of HA-NOG to stabilize HIF-1α.

#### 2.3.2. Expression of HIF-1 Target Genes

**Figure 4:**
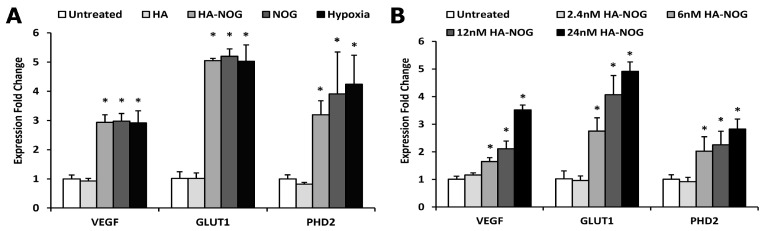
Relative mRNA expression levels of HIF-1 target genes vascular endothelial growth factor (VEGF), glucose transporter 1 (GLUT1), and prolyl hydroxylase domain 2 (PHD2). Normal human dermal fibroblasts (NHDFs) were cultured with (**A**) hyaluronic acid (HA, 24 nM), HA with conjugated N-oxyalylglycine (HA-NOG, 24 nM), NOG (10 μM), hypoxia (1% O_2_), and untreated control and (**B**) HA-NOG (2.4 nM, 6 nM, 12 nM, and 24 nM) and untreated control for 6 h. * identifies *p* < 0.05 relative to untreated control.

In the physiological hypoxia response, stabilized HIF-1α joins with the HIF-1β subunit, binds to HRE domains, and activates transcription of a wide range of hypoxia-responsive genes. Therefore, we next investigated mRNA expression levels of VEGF, glucose transporter 1 (GLUT1), and prolyl hydroxylase domain 2 (PHD2); these three direct targets of HIF-1 that mediate the hypoxia response by activating endothelial cells, increasing glucose transport, and putatively initiating negative feedback [11,47,48]. Expression of each target gene significantly increased for HA-NOG, NOG, and hypoxia conditions when compared to the HA and untreated controls (Figure 4A). VEGF, GLUT1, and PHD2 all exhibited 3–5 fold increases in expression for all three conditions. Consistent with qualitative observations of HIF-1α stabilization, there were no significant differences observed between HA-NOG, NOG, and hypoxia-positive control for any of the target genes, further confirming the ability of the HA-NOG macromolecular prodrug to activate the hypoxia response. We also investigated the effect of HA-NOG concentration on target gene expression. mRNA expression increased with increasing HA-NOG in a dose-dependent manner and was significantly higher for all targets at 6 nM, 12 nM, and 24 nM (2.5 μM, 5 μM, and 10 μM NOG, respectively) relative to the untreated control (Figure 4B). Expression levels were not significantly different for 2.4 nM (1 μM NOG) HA-NOG.

As noted above, other studies investigating NOG-based activation of HIF-1α have used the DMOG derivative based on its expected higher cell permeability. Interestingly, DMOG has been used at substantially higher concentrations than the 10 μM concentration of NOG used as a free drug or prodrug conjugate in the present study. In one of the earliest studies, Jaakkola et al. tested 0.1 and 1 mM DMOG in cell-based assays and found that 1 mM substantially increased HIF-1α expression detectable by Western blot [8]. More recently, several reports have investigated the dose-dependent effects of DMOG on HIF-1α stabilization and activation of target gene mRNA expression in various types of mesenchymal stem cells [49,50]. Responses consistently increased with increasing DMOG concentration and were significantly higher at 0.5 and 1 mM compared to 0.2 mM or untreated. Therefore, NOG is able to activate HIF-1 target gene expression at levels not significantly different from hypoxia positive control at two orders of magnitude lower concentration (10 μM) than that at which DMOG is conventionally used (1 mM).

#### 2.3.3. Endothelial Cell Tubulogenesis

**Figure 5:**
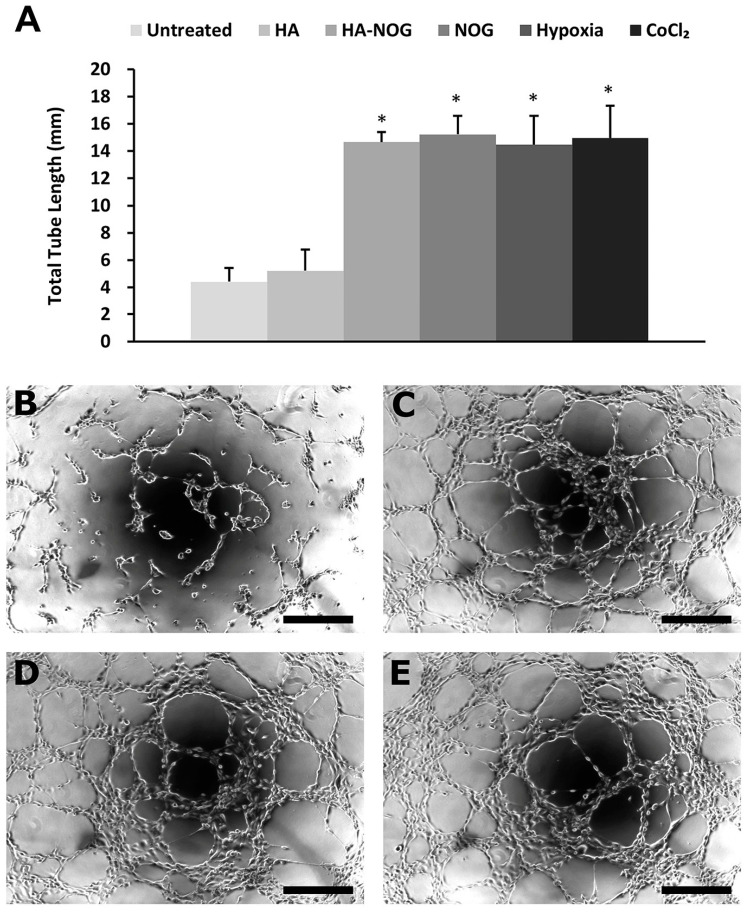
Tubulogenesis assay with human umbilical vein endothelial cells (HUVECs). (**A**) Total tubule length of HUVECs cultured with conditioned medium derived from normal human dermal fibroblasts (NHDFs) treated with hyaluronic acid (HA, 24 nM), HA with conjugated N-oxalylglycine (HA-NOG, 24 nM), NOG (10 μM), hypoxia (1% O_2_), CoCl_2_ (300 mM), and untreated control. (**B**–**E**) Representative images of HUVEC tubulogenesis during culture with conditioned medium derived from NHDFs treated with HA (**B**), HA-NOG (**C**), NOG (**D**), and (**E**) hypoxia. * identifies *p* < 0.05 relative to untreated control. Scale bar = 200 μm.

HIF signaling, whether activated directly by hypoxia or indirectly by exogenous stimuli such as NOG, culminates in the release of pro-angiogenic growth factors that act on surrounding cells to initiate angiogenesis. In these studies, we used endothelial cell tubulogenesis as an in vitro angiogenesis assay to investigate the presence of pro-angiogenic growth factors in a conditioned medium from NHDFs cultured in the presence of HA-NOG and various control conditions [51,52]. Total tubule length was significantly increased in human umbilical vein endothelial cells (HUVECs) cultured with conditioned medium from NHDFs treated with HA-NOG, NOG, CoCl_2_, and hypoxia relative to HA or untreated (Figure 5A). In addition, there were no significant differences among the HA-NOG and NOG groups and the CoCl_2_ and hypoxia-positive control groups. Figure 5B–E show representative images of HUVEC cultures. In Figure 5B, HUVECs cultured with conditioned medium from NHDFs exposed to native HA showed limited cell-cell connections that only included a few cells. In contrast, cultures treated with conditioned medium from NHDFs exposed to HA-NOG, NOG, and hypoxia (Figure 5C, Figure 5D, and Figure 5E, respectively) exhibited substantially increased polarization, cell–cell contact, and formation of elongated tubules. These results verify that HA-NOG activates the HIF-1 signaling pathway, leading to the secretion of pro-angiogenic signaling molecules that can activate endothelial cells to initiate therapeutic angiogenesis.

### 2.4. Cytotoxicity and Off-Target Effects

In addition to its central role in oxygen-sensing, HIF-1 activity can also be regulated by extracellular growth factors and cytokines and the activity of other intracellular signaling pathways [53,54]. Additionally, the HIF PHDs and FIH are members of a large family of 2-oxoglutarate-dependent dioxygenases including prolyl-4-hydroxylases critical for collagen synthesis and histone demethylases involved in chromatin modification and transcriptional regulation [3,21]. Therefore, exogeneous activation of the HIF pathway, particularly through inhibition of PHDs, may have detrimental effects on cell health and function. As a first step towards assessing safety, we investigated the effect of HA-NOG on NHDF metabolic activity and the accumulation of insoluble collagen matrix.

**Figure 6:**
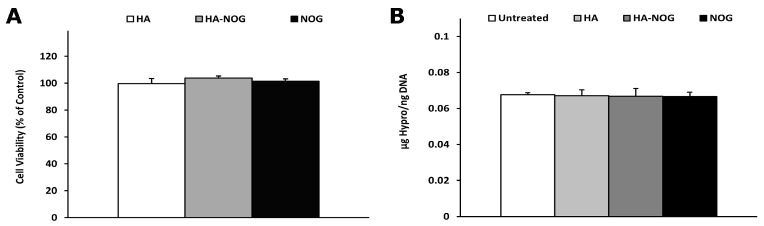
Metabolic activity (**A**) and hydroxyproline content (**B**) of NHDFs cultured in the presence of hyaluronic acid (HA, 24 nM), HA with conjugated N-oxalylglycine (HA-NOG, 24 nM), NOG (10 μM), and untreated control for 5 and 14 days, respectively.

After 5 days in culture, metabolic activity measured by the Alamar Blue assay was not significantly different between HA-NOG and NOG and untreated and HA-treated negative control groups (Figure 6A). Similarly, analysis of hydroxyproline content showed that there was no significant change for any experimental condition when compared to the untreated and HA-treated controls (Figure 6B). In addition, there were no significant differences in DNA content among the treatment groups measured for normalization of hydroxyproline content (Figure S2). Collectively, the metabolic activity and DNA content data indicate that exposure to HA-NOG does not adversely affect cell viability or proliferation. This contrasts with previous studies using DMOG that observed inhibition of cell proliferation [49,50]. The hydroxyproline analysis indicates that HA-NOG, at least at the concentration used in these studies, does not interfere with collagen synthesis and extracellular deposition. This is perhaps surprising, given that previous studies have shown NOG to be an effective inhibitor of prolyl hydroxylase domains (P4H), which is responsible for the formation of hydroxyproline, which is essential for the formation of the collagen triple helix [55]. Recently, Bentovim et al. investigated the mechanisms underlying collagen synthesis in the hypoxic environment of cartilaginous growth plates [56]. They found that hypoxia increased the mRNA expression of the three subunits that comprise P4H, as well as confirming the presence of HRE in the promoter of the pyruvate dehydrogenase kinase 1 (Pdk1) gene. They proposed a model for how growth plate chondrocytes achieve collagen synthesis under hypoxia that included increasing both the enzyme and substrate for proline hydroxylation through HIF-1-dependent increased P4H expression and Pdk1-mediated inhibition of the tricarboxylic acid cycle to reduce cellular oxygen consumption. This model offers one potential explanation for how HA-NOG is able to stabilize HIF-1α and initiate pro-angiogenic signaling without interfering with collagen metabolism. Although the exact mechanism remains uncertain, our results suggest that HA-NOG can activate the HIF-1 pathway without negatively impacting cellular health or an essential and closely related metabolic pathway.

## 3. Conclusions

N-oxalylglycine-conjugated hyaluronic acid (HA-NOG) was successfully synthesized as a macromolecular prodrug that released NOG by cell-mediated enzymatic degradation. The conjugate activated the HIF signaling pathway in fibroblasts, resulting in pro-angiogenic gene expression and secretion of soluble factors that activate endothelial cells without altering other cellular metabolic processes like proliferation and collagen synthesis. These results show that HA-NOG can serve as a safe and effective method for local, sustained release of NOG for treatment of ischemic pathology or support to tissue regeneration. In future studies, HA-NOG will be combined with the previously developed HA-DXM in hydrogel networks to coordinate osteogenic/angiogenic signaling essential to bone formation and regeneration.

## 4. Materials and Methods

### 4.1. Materials

Hyaluronic acid (HA, sodium salt, MW 1.5 MDa) was purchased from LifeCore Biomedical (Chaska, MN, USA). Carbonyldiimidazole (CDI), triethylamine (TEA), anhydrous dimethylsulfoxide (DMSO), 4-(dimethylamino)benzaldehyde (DMAB), 2-propanol, hydrochloric acid (HCl), sodium hydroxide (NaOH), citric acid monohydrate, anhydrous sodium acetate, glacial acetic acid, Chloramine-T, and ascorbic acid 2-phosphate (AA2P) were purchased from Sigma-Aldrich (St. Louis, MO, USA). Tetrabutylammonium bromide (TBA-Br) and Dowex 50WX8 200−400(H) were purchased from Alfa Aesar (Ward Hill, MA, USA). N-oxalylglycine (NOG) was purchased from Cayman Chemical (Ann Arbor, MI, USA). Normal human dermal fibroblasts (NHDFs), human umbilical vein endothelial cells (HUVECs), and endothelial cell growth medium (EGM-2) were purchased from Lonza (Walkersville, MD, USA). Dulbecco’s modified Eagle medium (DMEM), growth factor reduced Matrigel, and bovine growth serum (BGS) were purchased from Corning Life Sciences (Tewksbury, MA, USA). Medium 200PRF was purchased from Thermo Fisher (Waltham, MA, USA).

### 4.2. Synthesis and Characterization

#### 4.2.1. HA-TBA Synthesis

Dowex ion-exchange resin (3.0 g) was mixed with excess TBA-Br (5.0 g) for 1 h, washed, and then added to sodium hyaluronate (250 mg) dissolved in 50 mL of distilled water. After 3 h, the mixture was centrifuged (3 min at 4000 rpm), and the supernatant was collected and freeze-dried to obtain HA-tetrabutylammonium salt (HA-TBA).

#### 4.2.2. HA-NOG Conjugate

N-oxalylglycine-conjugated hyaluronic acid (HA-NOG) was prepared in two steps: First, the terminal carboxyl group of N-oxalylglycine (NOG) was activated with 1,1′-carbonyldiimidazole (CDI). The reaction was performed at two molar ratios of NOG to the HA disaccharide (0.85:1 and 1.7:1), corresponding to 10 and 20 mg NOG, respectively. NOG (10 mg, 0.068 mmol or 20 mg, 0.136 mmol) was dissolved in 2 mL DMSO, and TEA (45.48 mL, 0.326 mmol) was added. CDI (48.5 mg, 0.299 mmol) was dissolved in 2 mL DMSO, added dropwise to the NOG/TEA solution, and stirred for 2 h. In the second step, the activated NOG was conjugated to the hydroxyl groups of HA-TBA to obtain HA-NOG. HA-TBA (50 mg, 0.080 mmol) was dissolved in 20 mL DMSO and stirred for 24 h. The activated NOG/CDI solution was slowly added to the dissolved HA-TBA and stirred for 48 h in the dark. Sodium chloride (2.0 M) was added to the reactant solution at a 10% volume ratio for the exchange of TBA^+^ by Na^+^ ions. The solution was then purified by dialysis against ultrapure MilliQ water in 12–14 kDa MWCO tubing for 72 h, filtered (0.45 mm cellulose acetate filter), and recovered by lyophilization. The structure of HA-NOG was analyzed by ATR-FTIR using a Thermo-SpectraTech Foundation Series Endurance Diamond ATR (Thermo-Nicolet Magna 550, Thermo Fisher, Waltham, MA, USA) and compared to native HA. To measure the degree of substitution (DS), HA-NOG was hydrolyzed using 0.1 N sodium hydroxide (NaOH) at 60 °C for 1 h, and the pH neutralized with 0.1 N hydrochloric acid (HCl) and diluted with PBS to a final concentration of 2 mg/mL. The concentration of NOG was measured by UV absorbance at 220 nm using a NanoDrop 2000c UV-Vis Spectrophotometer (Thermo Fisher, Waltham, MA, USA) and a standard curve of NOG serially diluted in a solution of 2 mg/mL alkaline hydrolyzed HA. The DS of conjugated NOG was calculated as mg NOG/100 mg HA-NOG. A representative standard curve of absorbance at 220 nm as a function of NOG concentration in hydrolyzed native HA and absorbance spectra for NOG in water, hydrolyzed HA, NOG in hydrolyzed HA, and hydrolyzed HA-NOG are included in the Supplemental Information (Figure S3).

#### 4.2.3. Enzymatic Release of NOG from HA-NOG Conjugate

NOG release from soluble HA-NOG was evaluated in physiological buffer alone and with the addition of hydrolytic enzymes such as hyaluronidase (Hase) and esterase (Ease). HA-NOG was incubated in 0.1 M PBS (pH 7.4) solution, 0.1 M PBS containing Ease (5 U/mL), and 0.1 M PBS containing Ease and Hase (5 U/mL each) at 37 °C. Samples (100 mL) of each solution were removed each day for 7 days and stored at −20 °C until analysis. Samples (20 mL) were treated with methanol (80 mL), centrifuged, and filtered using a 0.2 mm syringe filter. The concentration of released NOG was measured by UV spectrophotometry, as described above.

### 4.3. Evaluation of Angiogenic Bioactivity

#### 4.3.1. Cell Culture

Cell culture for in vitro analysis was performed using NHDFs or HUVECs. NHDF cell culture medium consisted of DMEM/F-12 50/50 with L-glutamine (Thermo Fisher, Waltham, MA, USA) supplemented with bovine growth serum (BGS, Hyclone, Logan, UT, USA) and penicillin/streptomycin. HUVECs were cultured in EGM-2 endothelial cell growth medium.

#### 4.3.2. HIF-1α Stabilization

NHDF cells were seeded at 20,000 cells/well in 96-well plates and cultured at 37 °C for 24 h. The medium was exchanged, and the cells were cultured under the following experimental conditions (n = 4 wells/group): untreated, HA (24 nM), HA-NOG (24 nM, equivalent to 10 μM NOG based on DS calculations), NOG (10 μM), cobalt chloride (CoCl_2_, 300 mM), and hypoxia (1% O_2_). After 6 h, the plates were immediately put on ice and fixed with methanol. Methanol was removed, and staining media (95% PBS w/o Ca^+2^/Mg^+2^, 5% BGS, 0.1% NaN_3_) was added for blocking. HIF-1α primary antibody (Novus Biologicals, H1alpha67) at 1:100 dilution in staining media was added to the wells for 60 min. Wells were rinsed with staining media and incubated with goat anti-mouse IgG DyLight 488 secondary (Abcam, Cambridge, UK, 1:220 dilution in staining medium) for 60 min in the dark. Wells were rinsed three times, and DAPI dihydrochloride (Invitrogen, Carlsbad, CA, USA, 300 nM) counterstain was applied for 5 min. The wells were rinsed and imaged using an inverted fluorescence microscope (Zeiss Axiovert 200, Zeiss, Oberkochen, Germany).

#### 4.3.3. Expression of HIF-1 Target Genes

Using NHDFs, two studies were performed to test the ability of HA-NOG to increase mRNA expression of HIF-1α target genes and the dose dependence of this response. NHDFs were seeded (300,000 cells/well) in 6-well plates, cultured at 37 °C for 24 h, and then the medium exchanged. In the first study, cells were cultured under the following experimental conditions for 6 h (n = 3 wells/group): untreated, HA (24 nM), HA-NOG (24 nM), NOG (10 μM), and hypoxia (1% O_2_). In the second study, cells were cultured for 6 h with varying concentrations of HA-NOG (2.4 nM, 6 nM, 12 nM, and 24 nM) and untreated control (n = 3 wells/group). Cells were lysed with TRIzol Reagent (Ambion, Austin, TX, USA), and the aqueous phase was transferred to RNeasy columns (Qiagen, Germantown, MD, USA) for purification. Isolated RNA was treated with DNase I using the TURBO DNA-free kit (Invitrogen, Carlsbad, CA, USA). A Take 3 microplate reader (Biotek Instruments, Winooski, VT, USA) was used to determine the quantity and quality of isolated RNA by UV absorbance. Purified RNA (1 μg) was reverse transcribed with a High Capacity cDNA RT kit (Applied Biosystems, Waltham, MA, USA), and real-time qRT-PCR was performed using PowerUp SYBR Green Master Mix (AB) with custom sense and anti-sense primers (0.5 mM, Table 1) for vascular endothelial growth factor (VEGF), glucose transporter 1 (GLUT1), prolyl hydroxylase domain 2 (PHD2), and β-2 microglobulin (β2MG) as an internal standard, using a Rotor-Gene Q thermal cycler (Qiagen, Germantown, MD, USA). Relative mRNA expression levels were quantified by the 2^−ΔΔCt^ method, with results expressed as relative fold changes [57].

#### 4.3.4. Tubulogenesis Assay

NHDFs were seeded (300,000 cells/well) in 6-well plates with low-serum DMEM (1% serum) and incubated at 37 °C for 24 h. The medium was exchanged, and the cells were cultured under the following experimental conditions for 24 h (n = 4 wells/group): untreated, HA (24 nM), HA-NOG (24 nM), NOG (10 μM), hypoxia (1% O_2_), and CoCl_2_ (300 mM). Conditioned cell culture medium (5 mL) was harvested and stored at −80 °C for later use. HUVECs were seeded (20,000 cells/well) in a growth factor-reduced Matrigel-coated 96-well plate with low-serum Medium 200PRF (1% serum) (100 μL). After 3 h for attachment, the medium was replaced with the conditioned media (100 μL) obtained from NHDFs cultured under various experimental conditions, and cells were cultured for 6 h to allow in vitro tubulogenesis. HUVECs were fixed and imaged using a light microscope, and the total tube length was measured with ImageJ 1.52.

### 4.4. Cytotoxicity of Off-Target Effects

#### 4.4.1. Metabolic Activity Assay

NHDFs were seeded (50,000 cells/well) in 24-well plates and cultured for 24 h, then the medium was exchanged, and cells were cultured under the following experimental conditions for 5 days (n = 4 wells/group): untreated, HA (24 nM), HA-NOG (24 nM), and NOG (10 μM). AlamarBlue reagent (Thermo Fisher) was added in a volume equal to 1/10th the volume of the cell culture media in each well. After 12 h, fluorescence readings (555 nm excitation, 590 nm emission) were taken using a Biotek Synergy 4 plate reader (Thermo Fisher, Waltham, MA, USA).

#### 4.4.2. DNA and Hydroxyproline Analysis

Porous Tecoflex polyurethane sponges were fabricated as previously described [58]. NHDFs were seeded (2.4 × 10^6^ cells/scaffold) on fibronectin-coated Tecoflex sponges, cultured 24 h, and then the medium was exchanged and the materials cultured under the following experimental conditions (n = 4 samples/group): untreated, HA (24 nM), HA-NOG (24 nM), and NOG (10 μM). AA2P (1 mM) was added every 3 days with the changing of the cell culture media to ensure proper collagen formation. After 14 days, the sponges were rinsed with PBS and stored at −80 °C for 24 h. The sponges were subjected to repeated freeze/thaw cycles (−80 °C/37 °C) to lyse the cells. DNA was then solubilized and measured using Hoechst 33258 (Millipore Sigma, St. Louis, MO, USA), as previously described [59,60]. Briefly, a standard curve was made from freeze/thaw lysed cell pellets (0.1, 0.25, 0.5, 1.0, and 2.0 × 10^6^ cells). Lysed standard and sponge samples were treated with cold 10 mM EDTA, pH 12.3 DNA solubilization buffer (1.4 mL), incubated for 20 min at 37 °C, neutralized with 1M potassium phosphate (0.1 mL), and placed on ice. Hoechst 33258 DNA-binding dye (200 ng/mL, 1.5 mL) was added, 200 mL samples were transferred to a 96-well plate, and fluorescence was measured at 350 nm excitation, 455 nm emission using a Biotek Synergy 4 plate reader.

The Tecoflex substrates and retained insoluble collagen were then transferred to glass test tubes, and 1 mL of 12N HCl was added to each. Samples were hydrolyzed at 120 °C for 3 h, cooled for 20 min, transferred to new tubes, and centrifuged at 10,000 rpm for 3 min. Further, 20 mL of each supernatant were transferred to a 96-well plate and dried in a vacuum oven for 12 h. Hydroxyproline content was assayed as previously described [61]. Briefly, samples were mixed with 100 μL of Chloramine-T solution (0.05 M Chloramine-T in 74% *v*/*v* H_2_O, 26% *v*/*v* 2-porpanol, 0.629 M NaOH, 0.140 M citric acid monohydrate, 0.453 M anhydrous sodium acetate, and 0.112 M glacial acetic acid) at room temperature for 20 min and then 100 μL of Ehrlich’s solution (1 M DMAB in 30% *v*/*v* HCl and 70% *v*/*v* 2-propanol) was added and heated at 65 °C for 20 min. The 96-well plate was immediately put on ice for 5–10 min to quench the reaction. A standard curve was prepared by serial dilution from a 400 μg/mL hydroxyproline sample. Absorbance readings were taken at 558 nm using a Biotek Synergy 4 plate reader.

### 4.5. Statistical Analysis

Statistical comparisons were performed using one-way analysis of variance (ANOVA). *p*-values less than 0.05 were considered significant. Data are expressed as mean +/− standard deviation with significant differences marked as * (*p* < 0.05).

## Figures and Tables

**Table 1:** Primer sequences used in real-time RT-PCR.

Gene	Forward Primer (5′-3′)	Reverse Primer (5′-3′)	GeneBank No.	Product Size (bp)
*VEGF*	CCTTGCCTTGCTGCTCTACC	ACCAGGGTCTCGATTGGATG	NM_001171630	144
*PHD2*	TGTTATCCGGGCAATGGAAC	AAACTGGGCTTTGCCTTCTG	NM_022051	156
*GLUT1*	ACTCTTCAGCCAGGGTCCAC	CGTAGGGACCACACAGTTGC	NM_006516	119
*β2MG*	TGTGCTCGCGCTACTCTCTC	CGGATGGATGAAACCCAGAC	NM_004048	137

All primer sequences were designed based on gene sequences obtained from the respective GeneBank numbers using Primer-Blast (http://www.ncbi.nih.gov/tools/primer-blast/ accessed on 10 January 2017).

## Data Availability

The data presented in this study are available on request from the corresponding author.

## Supplementary Materials

The following supporting information can be downloaded at: https://www.mdpi.com/article/10.3390/gels11010027/s1, Figure S1. Immunocytochemical analysis of HIF-1a (green) and DAPI nuclear stain (blue) in normal human dermal fibroblasts (NHDFs) cultured under untreated (normoxia) condition and treatment with 300 mM CoCl_2_. Scale bar = 200 μm; Figure S2. Total cellular DNA content extracted from each substrate; Figure S3A. Standard curve for UV absorbance at 220 nm as a function of NOG concentration; Figure S3B. Absorbance spectrum of NOG (50 μg/mL) in water; Figure S3C. Absorbance spectrum of hydrolyzed native HA; Figure S3D. Absorbance spectrum of NOG (250 μg/mL) in hydrolyzed native HA; Figure S3E. Absorbance spectrum of hydrolyzed HA-NOG.
