# Supplementary material for: N-Oxalylglycine-Conjugated Hyaluronic Acid as a Macromolecular Prodrug for Therapeutic Angiogenesis
Source: Gels. 2025 Jan 2;11(1):27. doi: 10.3390/gels11010027 (PMC11765021; doi:10.3390/gels11010027)
Supplement: Supplementary file 1 [file gels-11-00027-s001.zip › gels-3275216-supplementary.pdf]

Supplemental Information

2.3.1 HIF-1α stabilization

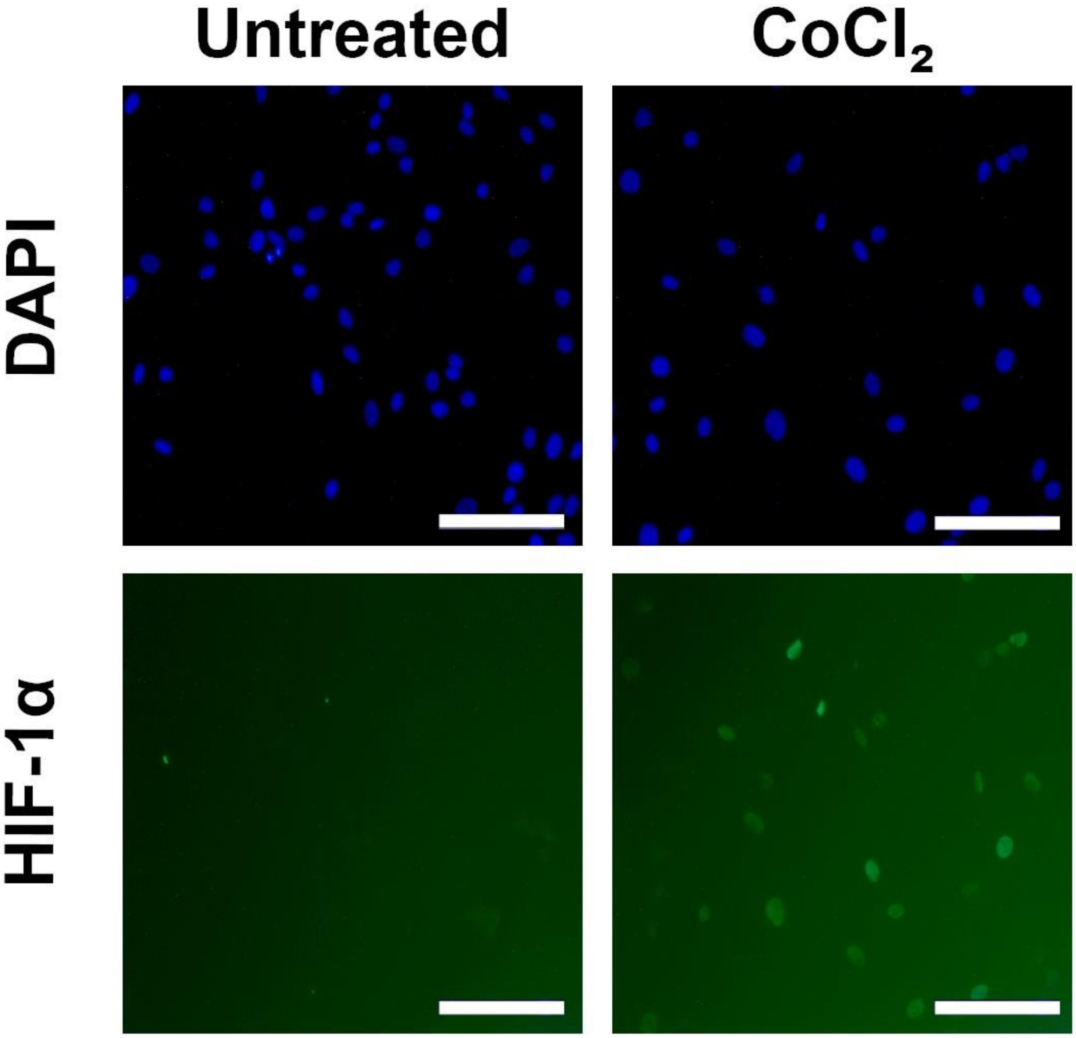

Figure S1. Immunocytochemical analysis of HIF-1a (green) and DAPI nuclear stain (blue) in normal human dermal fibroblasts (NHDFs) cultured under untreated (normoxia) condition and treatment with 300 mM $CoCl_2$. Scale bar = 200 μm.

2.4 Cytotoxicity and Off-Target Effects (DNA content)

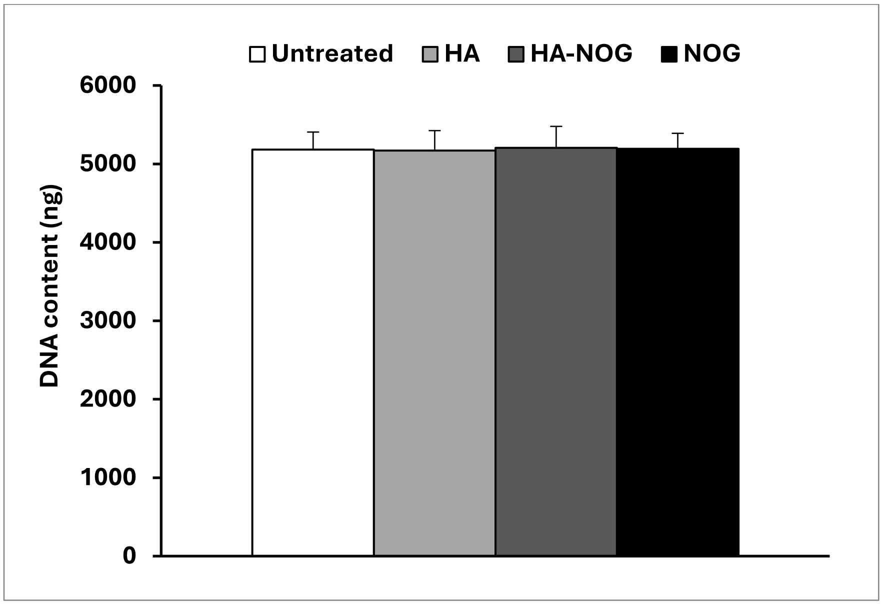

Figure S2. Total cellular DNA content extracted from each substrate.

4.2.2 HA-NOG conjugate

Hydrolyzed hyaluronic acid (HA), N-oxalylglycine (NOG), and NOG-conjugated HA (HA-NOG) were prepared as described in materials and methods. Supplemental Figure 3A shows a representative standard curve of absorbance at 220 nm as a function of NOG concentration diluted in hydrolyzed HA. Supplemental Figure 3B shows a representative absorbance spectrum for NOG prepared in water. Supplemental Figure 3C shows a representative absorbance spectrum of hydrolyzed native HA. This was the background subtracted from all standards and experimental samples. Supplemental Figure 3D shows a representative absorbance spectrum of NOG diluted in hydrolyzed HA. Supplemental Figure 3E shows a representative absorbance spectrum of hydrolyzed HA-NOG.

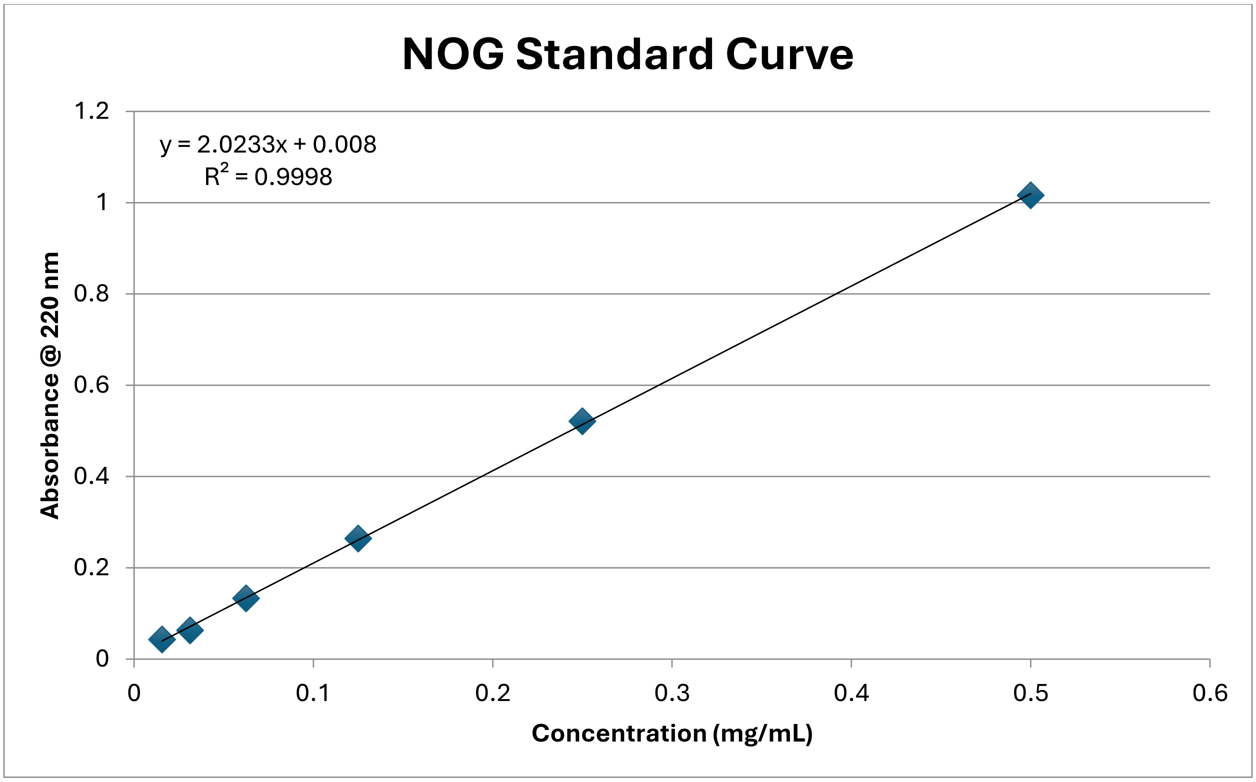

Figure S3A. Standard curve for UV absorbance at 220 nm as a function of NOG concentration.

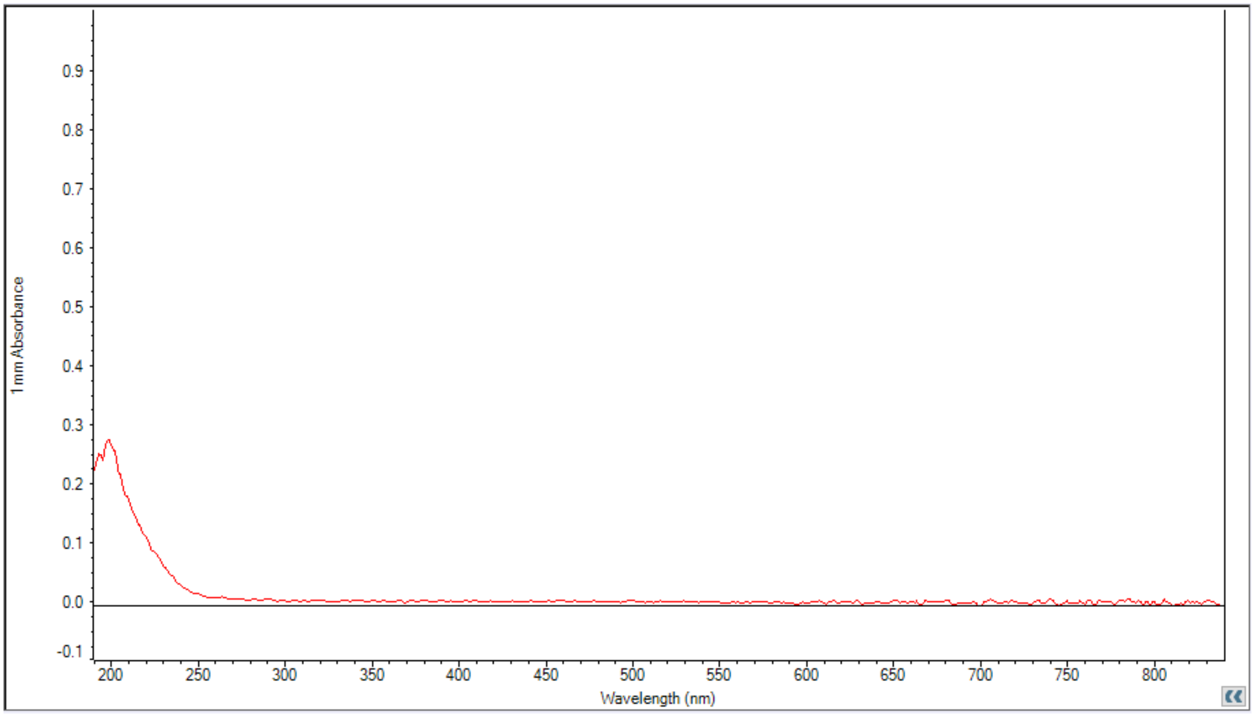

Figure S3B. Absorbance spectrum of NOG (50 µg/ml) in water.

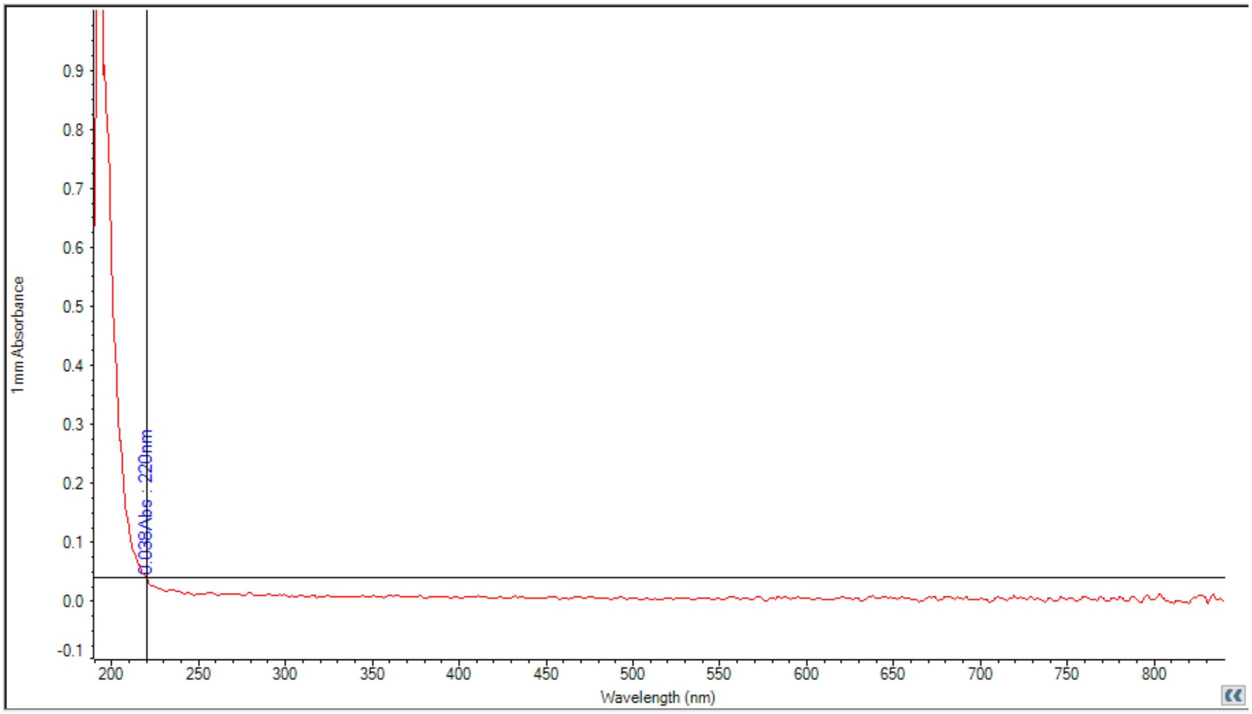

Figure S3C. Absorbance spectrum of hydrolyzed native HA.

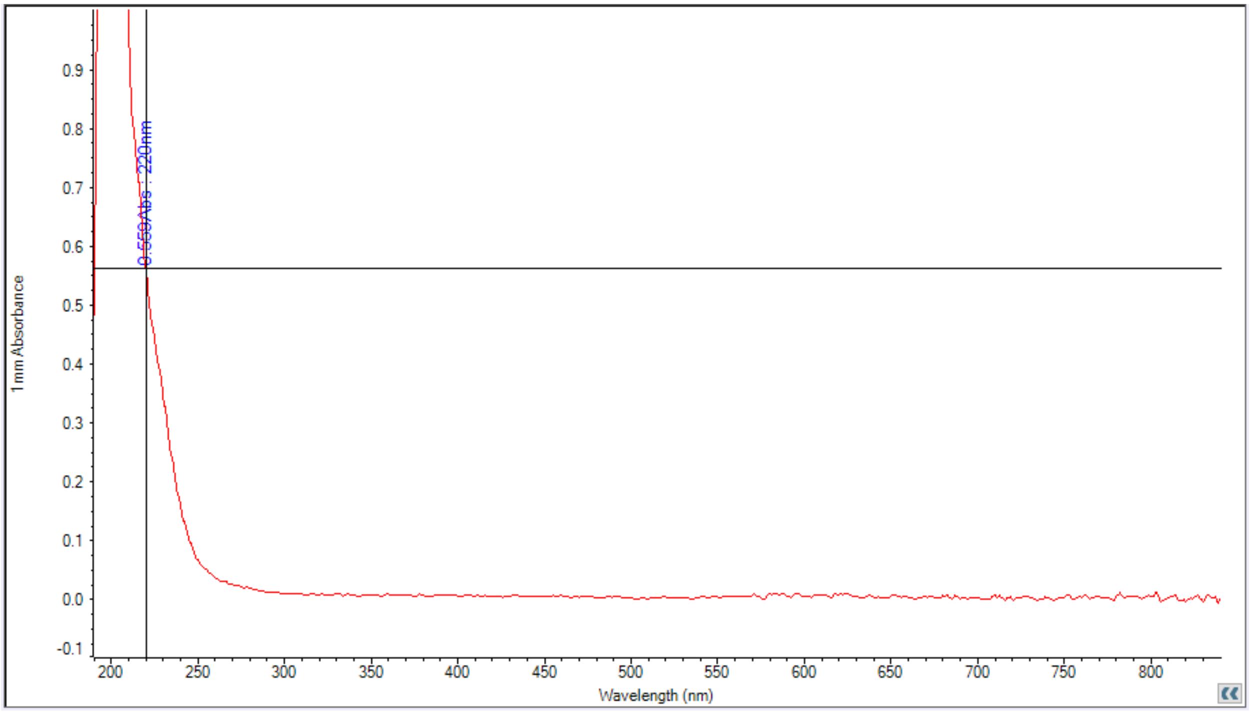

Figure S3D. Absorbance spectrum of NOG (250 µg/ml) in hydrolyzed native HA.

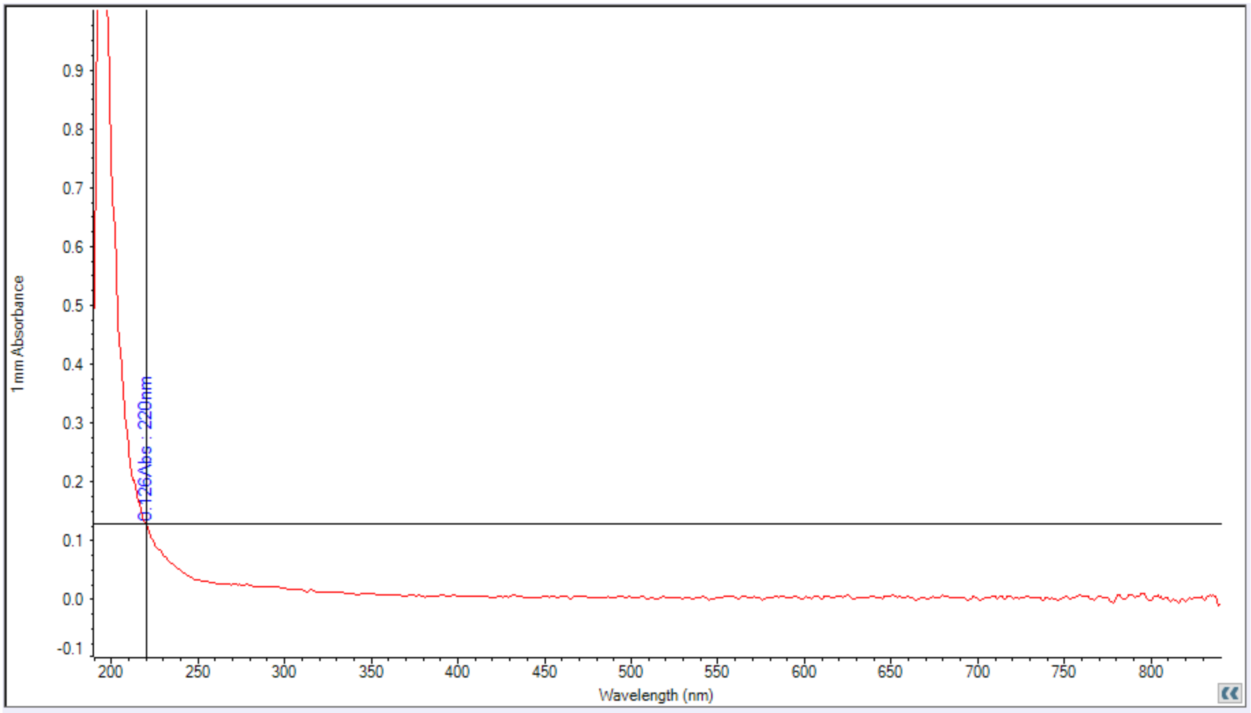

Figure S3E. Absorbance spectrum of hydrolyzed HA-NOG.
